# Perspectives on smoking cessation in the oncology environment: insights from brazilian patients and healthcare providers

**DOI:** 10.1590/1980-549720250046

**Published:** 2025-08-08

**Authors:** Raquel Descie Veraldi Leite, Ricardo Ribeiro Gama, Fabiana de Lima Vazquez, Gabriela Beltrami Massarão, Raiany Carvalho, Renan de Jesus Teixeira, Vinicius de Lima Vazquez, Irene Tami-Maury

**Affiliations:** I Hospital de Câncer de Barretos - Barretos (SP), Brazil.; II University of Texas Health Science Center at Houston - Houston, Texas, United States.

**Keywords:** Prevalence, Barriers, Healthcare professionals, Cancer patients, Smoking cessation, Prevalência, Barras, Profissionais de saúde, Pacientes com câncer, Parar de fumar

## Abstract

**Objective:**

The aim of this study was to identify barriers and facilitators to smoking cessation among Brazilian cancer patients, considering the perspectives of both patients and healthcare professionals at a leading oncology center in the country.

**Methods:**

A cross-sectional study was conducted, collecting smoking-related data from two groups: cancer patients and healthcare professionals at the Barretos Cancer Hospital (BCH) between 2019 and 2021. The questionnaire for healthcare professionals was adapted from the 2012 International Association for the Study of Lung Cancer and the Global Adult Tobacco Survey. For the cancer patient group, sociodemographic and clinical data were collected, along with the smoking history and consumption patterns of current smokers.

**Results:**

Among oncology patients, the prevalence of former smokers was found to be 37.4%, while current smokers accounted for 16.8%. Most current smokers exhibited low nicotine dependence and high motivation to quit. Key barriers reported by healthcare professionals in providing smoking cessation interventions included patient resistance (86.9%) and lack of training (64.5%). Furthermore, 52.9% of these professionals indicated that they had never discussed cessation strategies during consultations with smoking patients. Regression models revealed that physicians, compared to other professionals, were more likely to address, advise, and offer cessation treatment to smoking patients (p≤0.05).

**Conclusion:**

There is a need to enhance training on smoking cessation for healthcare professionals to improve clinical outcomes and survival rates among cancer patients.

## RESUMO

**Objetivo:**

Identificar barreiras e facilitadores para a cessação do tabagismo entre pacientes oncológicos brasileiros, considerando as perspectivas dos pacientes e dos profissionais de saúde em um centro de referência em tratamento oncológico no país.

**Métodos:**

Estudo transversal que coletou dados sobre tabagismo de dois grupos, pacientes com câncer e profissionais de saúde, do Hospital de Câncer de Barretos (HCB), durante o período de 2019 a 2021. O questionário aplicado ao grupo de profissionais de saúde foi adaptado da International Association for the Study of Lung Cancer de 2012 e do Global Adult Tobacco Survey. Para o grupo de pacientes oncológicos, foram coletados dados sociodemográficos e clínicos, além do histórico e padrões de consumo de tabaco dos pacientes fumantes.

**Resultados:**

Foi observada prevalência de 37,4% de ex-fumantes e 16,8% de fumantes atuais entre os pacientes oncológicos. A maioria dos fumantes atuais apresentou baixa dependência de nicotina e alta motivação para a cessação. As principais barreiras relatadas pelos profissionais de saúde para a oferta de intervenções antitabagistas incluíram resistência dos pacientes (86,9%) e falta de treinamento (64,5%). Além disso, 52,9% desses profissionais nunca discutiram estratégias de cessação durante as consultas com pacientes fumantes. Os modelos de regressão demonstraram que os médicos, em comparação com outros profissionais, são mais propensos a abordar, aconselhar e oferecer tratamento de cessação aos pacientes fumantes (p≤0,05).

**Conclusão:**

Esforços devem ser direcionados para aprimorar o treinamento sobre cessação do tabagismo para profissionais de saúde, contribuindo para melhorar os resultados clínicos e a sobrevivência dos pacientes com câncer.

## INTRODUCTION

Smoking is widely recognized as a risk factor for the development and progression of cancer. Studies show that continuing to smoke after a cancer diagnosis significantly increases the risk of disease recurrence and mortality^1^, and persistent smoking can significantly reduce the effectiveness of treatment and decrease the chances of cure^2^ ^,^ ^3^.

Patients who quit smoking after a cancer diagnosis improve their prognosis and overall health outcomes^1^ and despite the well-established benefits of smoking cessation, relapse rates among cancer survivors remain high^3^.

Evidence in the literature highlights significant challenges faced by cancer patients seeking to quit smoking. Barriers such as pain, high levels of nicotine dependence, anxiety, and depression can negatively impact this process^4^. Sociodemographic factors such as education, employment, and income are crucial to understanding smoking cessation. Furthermore, most smokers who attempt to quit do so without professional assistance or support^3^ ^,^ ^5^. Understanding tobacco use-related issues among cancer patients is essential to implementing effective smoking cessation measures and improving health outcomes for these patients^6^.

International studies have increasingly emphasized the critical role of healthcare professionals in the success of smoking cessation strategies among cancer patients^7^ ^,^ ^8^. However*, persistent* challenges remain, particularly related to the lack of preparedness among providers. This is often linked to a perceived deficiency in training and education on tobacco treatment. Such deficiency can generate insecurities - manifested as limited knowledge, skills, and confidence in delivering smoking cessation interventions - and has been widely reported in the literature^9^. Several studies have underscored the need to integrate structured tobacco treatment into oncology care to improve patient outcomes and enhance the ability of healthcare providers to screen, advise, and refer patients who smoke to appropriate treatment programs^10^ ^,^ ^11^ ^,^ ^12^. While these findings are well documented in high-income countries, research addressing this issue in the Brazilian oncology context remains limited. Therefore, additional studies are warranted to explore the extent to which these international patterns hold true in Brazil and to identify context-specific barriers and facilitators for effective smoking cessation within cancer care.

This study aimed to analyze the prevalence of smoking and the profile of cancer patients and healthcare professionals at a leading oncology center in Brazil, as well as to identify the main barriers and facilitators to smoking cessation from the perspective of healthcare professionals.

## METHODS

### Study design

This was a cross-sectional observational study conducted at the Barretos Cancer Hospital (BCH), a reference center for cancer treatment and prevention in Sao Paulo State, Brazil. The study included two distinct and independent groups: cancer patients and healthcare professionals, each assessed using specific inclusion criteria, recruitment strategies, and instruments.

### Cancer patients’ group

#### Population, inclusion criteria, and study sample

Cancer patients aged 18 years or older, undergoing treatment or follow-up at BCH between November 2016 and January 2019, and with an Eastern Cooperative Oncology Group (ECOG) performance status score of 2 or less were eligible for inclusion. The sample size was calculated based on a smoking prevalence of 14.7% among Brazilian adults^13^, with a 5% margin of error and a 95% confidence level, resulting in a minimum sample of 190 participants. To account for potential attrition, the final target was set at 210 patients. Between November 2016 and January 2019, 10,057 patients were diagnosed at BCH with primary tumors corresponding to the cancer types included in this study. Therefore, the final sample of 210 patients represents approximately 2.1% of the total eligible population during the study period. While the sample was not designed to be fully representative of the entire hospital cohort, it was probabilistically stratified by sex and cancer type to enhance internal validity and ensure adequate representation of the most prevalent cancer subtypes treated at the institution. Among female participants, the distribution was as follows: breast (50.5%), colorectal (17.1%), cervical (13.3%), thyroid (10.5%), and lung cancers (8.6%). Among male participants, the distribution was as follows: prostate (48.6%), colorectal (20.0%), lung (13.3%), gastric (11.4%), and oral/oropharyngeal cancers (6.7%).

#### Recruitment and data collection

Patients were approached and invited to participate in the study from the departments of breast, colon and rectum, cervix, head and neck, thoracic, urology, and upper digestive tract, BHC. A hybrid approach was adopted due to the COVID-19 pandemic. Of the 210 patients, 119 were interviewed in person and 91 by telemedicine. All interviews were conducted by the same trained researcher using standardized instruments.

#### Questionnaires

These included a *sociodemographic questionnaire* and collection of detailed data on primary and secondary cancers, including diagnostic data, tumor location, and ongoing treatment regimens; a *questionnaire addressing tobacco history and consumption patterns*, covering factors such as initiation of tobacco use, variety of products, and frequency of use; the *Fagerström Scale* ^*14*^, which is an instrument that measures the degree of dependence on nicotine specifically; and the *Richmond Scale* ^*15*^, which measures motivation to quit smoking.

Also included were the *Rapid Assessment of Physical Activity (RAPA*)^16^ questionnaire, which measures participants’ physical activity, and the *Hospital Anxiety and Depression Scale (HADS)* ^*17*^, which measures the presence of hospital-related anxiety and depression.

### Healthcare professionals’ group

#### Population, inclusion criteria, and study sample

The study population consisted of healthcare professionals who were working at BCH between February and June 2019. The sample of healthcare professionals consisted of 169 of the total 770 healthcare providers who directly care for cancer patients at BCH. The sample was obtained through convenience sampling, and no sample size calculation was performed for this group.

#### Recruitment

Recruitment took place from February to June 2019 and was promoted via emails, banners, website posts, and cards distributed throughout departments. Participation was voluntary, with access to the survey provided via an emailed link, with follow-up emails sent to increase participation rates. After providing informed consent, participants were able to complete an online questionnaire and complete the survey.

#### Online questionnaire

A semi-structured questionnaire was used that included:

1. Personal and occupational data (gender, age, professional qualification, and department);
2. Smoking prevalence; and
3. Attitudes and behavior related to tobacco use in the oncology setting (e.g., opinions on smoking in cancer patients, knowledge about tobacco use and its associated health risks, and perceived barriers to smoking cessation).

The assessment of attitudes and behavior was carried out through closed-ended questions with response options on a 5-point Likert scale, ranging from “strongly disagree” to “strongly agree.”

Additionally, to explore the main barriers that healthcare professionals perceive as interfering with the implementation of smoking cessation interventions for cancer patients, the questionnaire included the following statements, also evaluated on a 5-point Likert scale: difficulty in motivating patients to quit; belief that smoking cessation does not affect treatment or survival; lack of time to offer counseling; patient resistance; lack of training or experience; lack of resources or referral centers; treatment costs; limited availability of cessation medications in the public health system; tobacco use among healthcare professionals; and the marketing of various tobacco and e-cigarette products.

#### Data collection

The Qualtrics platform was used to collect data from these healthcare professionals. Qualtrics is a cloud-based system that facilitates the creation and secure distribution of web-based surveys. The survey was an anonymous self-administered survey hosted on this platform.

### Statistical analysis

Descriptive analysis (means, standard deviation, frequency, and proportions) was used to present the characteristics in both groups, of the cancer patients and healthcare professionals. To identify associations between the collected data, we used the Fisher’s or chi-square test. We used univariate logistic regression analysis with the intention of identifying factors associated with some questions; in addition, we also calculated the size of the effect of these analyses. The 5-point Likert scale responses were analyzed by combining “completely disagree,” “disagree,” or “no opinion” into a single category and “agree” or “strongly agree” into another group. Results were expressed with the level of significance set at p≤0.05. The minimum statistical power for both samples was set at 0.80. We performed statistical analysis with SPSS 21.0 software for Windows.

The prevalence of current smokers was determined based on those who reported having smoked 100 or more cigarettes in their lifetime and were smoking daily or occasionally at the time of the survey. Former smokers were defined as individuals who had smoked at least 100 cigarettes over their lifetime but had stopped smoking at the time of the survey.

The project was approved by the National Research Ethics Committee of the National Health Council under protocol 93691118.0.0000.5437. All participants signed the informed consent form before participating in the interview.

### Data Availability Statement

The data used in this study are available from the corresponding author upon reasonable request. Due to the sensitive nature of the information and institutional confidentiality policies, the dataset is not publicly available. Access may be granted upon approval by the relevant ethics committee and the signing of a confidentiality agreement, in accordance with institutional guidelines.

## RESULTS

Among 210 cancer patients who answered the tobacco use question, 45.8% were never-smokers, 37.4% former smokers, and 16.8% current smokers (Table 1). Never-smoking was more frequent among women and patients under 40 years of age. Cervical cancer had the highest proportion of current smokers; lung, mouth/oropharyngeal, and stomach cancers were most common among former smokers, while never-smokers predominated in thyroid, breast, and colorectal cancers.

**Table 1.** Sociodemographic and clinical characteristics reported by cancer patients’ group included in the study according to smoking status. Barretos Cancer Hospital, Brazil, 2019-2021.

Characteristics	Smoking status			Total, n	p-value
	Current smoker% (n)	Former smoker% (n)	Never smoker% (n)		
Smoking prevalence	16.8 (31)	37.4 (73)	45.8 (106)	210	-
Age (years old)					
21-40	6.7 (1)	6.7 (1)	86.7 (13)	205	0.049*
41-60	14.9 (13)	36.8 (32)	48.3 (42)		
>61	15.5 (16)	38.8 (40)	45.6 (47)		
Sex					
Female	12.4 (13)	21.9 (23)	65.7 (69)	209	<0.01*
Male	16.3 (17)	49 (51)	34.6 (36)		
Scholarity^†^					
Elementary school	19.7 (23)	34.2 (40)	46.2 (54)	206	0.13
High school	7.4 (4)	40.7 (22)	51.9 (28)		
Higher education	8.6 (3)	28.6 (10)	62.9 (22)		
Income^‡^					
Low income	22.7 (17)	26.7 (20)	50.7 (38)	206	0.18
Middle income	10.5 (13)	39.5 (49)	50 (62)		
High income	0 (0)	57.2 (4)	42.8 (3)		
Level of physical activity practice (RAPA)^§^					
Sedentary	18 (22)	37.7 (46)	44.3 (54)	210	0.23
Underactive	11.1 (1)	22.2 (2)	66.7 (6)		
Underactive (light activities)	7.9 (3)	26.3 (10)	65.8 (25)		
Underactive (regular)	4.3 (1)	47.8 (11)	47.8 (11)		
Active	22.2 (4)	27.8 (5)	50 (9)		
Cancer type					
Breast	13.2 (7)	15.1 (8)	71.7 (38)	209	<0.01*
Prostate	15.7 (8)	37.3 (19)	47.1 (24)		
Lung	21.7 (5)	73.9 (17)	4.3 (1)		
Cervical	28.6 (4)	35.7 (5)	35.7 (5)		
Stomach	16.7 (2)	66.7 (8)	16.7 (2)		
Colorectal	7.7 (3)	28.2 (11)	64.1 (25)		
Thyroid	9.1 (1)	9.1 (1)	81.8 (9)		
Oral and oropharynx	14.3 (1)	71.4 (5)	14.3 (1)		
Second primary tumor					
Yes	15.0 (3)	25 (5)	60.0 (12)	210	0.59
No	14.7 (28)	36.3 (69)	48.9 (93)		

Fisher’s or ꭓ^2^ test; Statistically significant values are denoted in bold; Values of total number of cancer patients responding differently to each question due to the questionnaire being self-administered and because it is possible to “skip” questions that they did not want to answer.

*p<0.05; ^†^Elementary school: Individuals who completed up to the 9th grade of formal education; high school: Individuals who completed up to the 12th grade (or 3rd year of high school); higher education: This category includes individuals who completed a higher education degree, meaning those who graduated from college or university; ^‡^Family income based on the 2021 Brazilian minimum salary (BMS) - R$1,100.00/low income (below 2 BMS); middle income (from 2 to 10 BMS); high income (above 2 BMS); ^§^Level of physical activity measured using the Rapid Assessment of Physical Activity (RAPA) scale. Sedentary: No regular physical activity or exercise; Underactive: Some physical activity, but below recommended levels; Underactive (light activities): Light activities (e.g., casual walking, household chores), insufficient for health improvement; Underactive (regular): Regular but moderate activity, below recommended guidelines; Active: Meets or exceeds the recommended levels of moderate-to-vigorous physical activity for health benefits.

Missing data, not all the variables add to the total: Age (5); sex (1); scholarity (4); cancer type (1).

Table 2 describes smoking patterns among current and former smokers. Smoking onset averaged 14.6 years of age. Former smokers: Median age - 43 years old (SD 12.1); Current smokers: Median age - 43.5 years old (SD 11.1). Former smokers reported a daily average of 24.1 cigarettes versus 9.2 for current smokers. Over half of the current smokers had low nicotine dependence (53.8%) and high motivation to quit (74.1%) (Table S1 - supplementary material).

**Table 2.** Information on the tobacco use of cancer patients’ group, such as age at smoking initiation, age at cessation, and length of tobacco use, in addition to the smoking history of former and current smokers. Barretos Cancer Hospital, Brazil, 2019-2021.

Smoking characteristics	Mean (SD)	Median (IQR)	Total, n
Age of smoking initiation (current smoking and ex-smoking patients)	14.6 (2.8)	14 (13-16)	103
Age of smoking cessation (ex-smoking patients)	42 (13.3)	44 (34-52)	74
Smoking period (current smoking patients)	34.2 (17.7)	37 (22-48)	26
Daily smoking load (ex-smoking patients)	24.1 (12.7)	20 (20-30)	74
Daily smoking load (current smoking patients)	9.2 (9)	10 (0-15)	28

Descriptive analysis. SD: Standard deviation; IQR: Interquartile range.

The analysis using the HADS revealed that 37.1% of the cancer patients exhibited anxiety components. In terms of depression, 14.8% of the patients showed depressive symptoms. The specific scores for each assessment are detailed in Table S2 - supplementary material.

The association between psychological components and levels of nicotine dependence among current smokers was assessed using Fisher’s exact test, and the results are presented in Table 3. It was observed that patients with moderate nicotine dependence were more likely to exhibit anxiety components (p=0.048).

**Table 3.** Association between psychological factors and nicotine dependence levels among current smoking cancer patients, assessed using Fisher’s exact test. Barretos Cancer Hospital, Brazil, 2019-2021.

Scale/Variable	Fagerstrom			n	p-value
	Low dependence n (%)	Moderate dependence n (%)	High dependence n (%)		
HADS					
With anxiety	42.9 (6)	90 (9)	50 (1)	26	0.048*
Without anxiety	57.1 (8)	10 (1)	50 (1)		

Fagerstrom Test for Nicotine Dependence (score: low dependence 1-4; moderate dependence 5-7; high dependence >8).

HADS: Hospital Anxiety and Depression Scale.

Fisher’s exact test (*p<0.05) was used due to the small sample size (n=26). All 26 cancer patients who were current smokers were included in this analysis. Statistically significant values are denoted in bold.

A total of 169 healthcare professionals responded to the survey, representing 24% of BCH’s workforce in 2019. Majority were female (58.4%), aged 21-30 years (40.0%), and physicians (51.3%). Overall, 65.6% devoted over 50% of their time to patient care. The smoking prevalence among professionals was 2.5%; 19% were former smokers and 78.5% had never smoked (Table S2 - supplementary material). We assessed how often professionals addressed smoking during patient encounters. Although 74.9% felt prepared to advise patients on tobacco risks, 52.9% reported never discussing cessation strategies. Regarding perceived barriers to cessation, 96.4% agreed that quitting smoking impacts treatment outcomes. The most cited barriers were patient resistance (86.9%), lack of training (64.5%), limited time for counseling or referrals (63.9%), and difficulty motivating patients (57.0%) (Table 4).

**Table 4.** Main barriers that interfere with smoking cessation interventions in cancer patients in the opinion of health professionals’ group. Barretos Cancer Hospital, Brazil, 2019-2021.

Barriers	Variables	Relative frequency (%)	Absolute frequency (n)	Total, n
Difficulty motivating patients to stop using tobacco products	Agree	57.0	61	107
	Disagree	43.0	46	
Waste of time - smoking cessation does not affect the treatment or survival of cancer patients or other chronic diseases	Agree	3.6	4	109
	Disagree	96.4	105	
Lack of time to provide anti-smoking counseling to the patient or referral to a smoking cessation center	Agree	36.1	39	108
	Disagree	63.9	69	
Patient resistance to treatment to stop smoking or to stop consuming tobacco products	Agree	86.9	93	107
	Disagree	13.1	14	
Lack of training or experience to offer and conduct tobacco control interventions	Agree	64.5	69	107
	Disagree	35.5	38	
Lack of available resources or reference centers for smoking cessation	Agree	57.0	61	107
	Disagree	43.0	46	
Treatment costs for quitting smoking	Agree	42.6	46	108
	Disagree	57.4	62	
Lack of availability of drugs for smoking cessation in public health	Agree	50.9	55	108
	Disagree	49.1	53	
Consumption of tobacco products by health professionals	Agree	37.4	40	107
	Disagree	62.6	67	
Marketing of a wide variety of tobacco products and electronic cigarettes as a less harmful alternative	Agree	43.5	47	108
	Disagree	56.5	61	

Values of the total number of health professionals responding differently to each question due to the questionnaire being self-administered and because it is possible to “skip” questions that they did not want to answer. Likert scale responses were analyzed by combining “completely disagree,” “disagree,” or “no opinion” into a single category (Disagree) and “agree” or “strongly agree” into another group (Agree).

Univariate logistic regression models were used to compare the likelihood of physicians performing smoking-related interventions with cancer patients against other professionals (Model 1) and nurses (Model 2). Frequency and odds ratios (ORs) for each action are presented in Table 5. In Model 1, physicians were significantly more likely than other professionals to: ask about smoking (OR 6.86, 95% confidence interval [CI] 2.92-16.14), inquire about quitting intentions (OR 3.74; 95%CI 1.72-8.16), provide cessation guidance (OR 3.57; 95%CI 1.59-8.02), and offer treatment or referrals (OR 2.85; 95%CI 1.11-7.35). In Model 2, compared to nurses, physicians were more likely to: ask about smoking (OR 8.06; 95%CI 2.46-26.42), assess intent to quit (OR 4.63; 95%CI 1.49-14.35), guide cessation (OR 3.73; 95%CI 1.26-11.05), and offer treatment or referrals (OR 8.33; 95%CI 1.04-66.87).

**Table 5.** Univariate analysis using the logistic regression method to assess the chances of health professionals always or most of the time asking their cancer patients about tobacco use of the health professionals’ group. Barretos Cancer Hospital, Brazil, 2019-2021.

Logistic regression model	Interventions			
	Ask cancer patients that whether they smoke or use tobacco products	Ask smoking patients that whether they intend to quit smoking	Guide cancer patient smokers to quit smoking	Offer treatment or refer patients for smoking cessation interventions
Model 1				
Total, n	n=116	n=116	n=116	n=113
ES (95%CI)	0.55 (0.17-0.92)	0.73 (0.34-1.10)	0.70 (0.32-1.08)	0.58 (0.19-0.96)
Other professionals^†^				
n	46	70	47	43
%	34.6	60.3	39.8	37.1
OR (95%CI)	-	-	-	-
Physicians				
n	70	69	71	70
%	60.3	58.5	60.2	62.9
OR (95%CI)	6.86 (2.92-16.14)*	3.74 (1.72-8.16)*	3.57 (1.59-8.02)*	2.85 (1.11-7.35)*
Model 2				
Total, n	n=86	n=88	n=89	n=86
ES (95%CI)	1.16 (0.57-1.71)	0.84 (0.31-1.36)	0.72 (0.19-1.25)	1.16 (0.59-1.73)
Nurses				
n	16	19	18	16
%	18.6%	21.6%	20.2%	18.6%
OR (95%CI)	-	-	-	-
Physicians				
n	70	69	71	70
%	81.4%	78.4%	79.8%	81.4%
OR (95%CI)	8.06 (2.46-26.42)*	4.63 (1.49-14.35)*	3.73 (1.26-11.05)*	8.33 (1.04-66.87)*

ES: Effect size (ES<0: adverse effect; 0.0≤ES≤0.1: no effect; 0.2≤ES≤0.4: small effect; 0.5≤ES≤0.7: intermediate effect; ES≥0.8: large effect); CI: Confidence interval; OR: Odds ratio.

*p<0.05; ^†^Others professionals include nurses, psychologists, physiotherapists, nurse technicians, auxiliary nurses, and radiotherapy technologists.

Univariate logistic regression analysis. Model 1 compares physicians to other professionals (psychologists, physiotherapists, nurse technicians, auxiliary nurses, and radiotherapy technologists); Model 2 compares physicians to nurses.

Response variable: Professionals who reported performing the intervention “always” or “most of the time.” Reference category: “Other professionals” for Model 1; “nurses” for Model 2. Model 1: No variables with statistically significant.

## DISCUSSION

This study illuminates the multifaceted interactions among smoking behaviors, specific cancer diagnoses, psychological comorbidities, and the healthcare professionals’ engagement with smoking cessation interventions, providing perspectives into challenges and opportunities within oncology care. By delving into these dimensions, we can better understand the nuances of integrating smoking cessation into oncology care.

In our study, 16.8% of the cancer patients were current smokers; 75.9% expressed interest in quitting within 6 months. These findings underscore the potential for targeted cessation interventions, aligning with Evans et al., who noted improved outcomes and reduced costs with integrated programs in oncology settings. The high interest in quitting highlights the need for tailored support to maximize clinical and economic benefits^18^. Though not statistically significant, in our study, majority of the current smokers have a low-income level, ex-smokers have a middle-income level, and no high-income patients smoked. These findings align with existing literature, which highlights that sociodemographic factors such as income and education play a critical role in determining smoking cessation success among cancer patients^19^. Validated scales showed that 74.1% of the smokers in our study had high motivation to quit (Richmond Test), while 53.8% displayed low physical nicotine dependence (Fagerström score). Similarly, Balduyck et al. reported that patients who quit smoking after lung cancer surgery experienced significant improvements in quality of life, particularly in physical functioning and overall well-being. These findings suggest that the high motivation and low dependence observed in our cohort could translate into substantial benefits if cessation efforts are effectively supported, reinforcing the critical role of cessation programs in improving both clinical outcomes and patients’ quality of life^20^. Additionally, the Global Adult Tobacco Survey (GATS, Brazil) identified links between lower nicotine dependence, lower education levels, and lower income^21^. Most participants showed no significant anxiety or depression levels, with 37.1% and 14.8%, respectively, according to the HADS. However, nicotine dependence was significantly associated with anxiety components (p=0.048).

The prevalence of smoking among cancer patients is consistently higher than in the general population^12^. Furthermore, studies highlight concerning relapse rates among cancer survivors who quit smoking at the time of diagnosis, emphasizing the importance of effective smoking cessation strategies and continuous support for patients^2^. Regarding the profile of smokers, a wide range of characteristics is observed, including demographic and clinical differences. Some studies identify specific risk factors associated with smoking in cancer patients, such as younger age, lower educational attainment, and the presence of comorbidities^11^. These insights are crucial for tailoring personalized smoking cessation interventions.

Notably, counseling for smoking cessation is more effective among individuals with lower nicotine dependence, resulting in reduced withdrawal symptoms and higher success rates^22^ ^,^ ^23^. Our study, consistent with Warren et al., found that 54.2% of cancer patients were current or former smokers, underlining the need for effective cessation support in this population^24^. Within our sample of current smoking oncology patients, over 70% were classified as sedentary per the RAPA scale. Sedentary behavior can hinder smoking cessation efforts, as regular physical activity has been shown to aid in cessation^25^. Furthermore, we observed a substantial percentage of never-smokers among patients aged up to 40 years, with 86.7% reporting no smoking experience. This underscores the success of public policies, such as the National Tobacco Control Policy (NTCP), implemented in Brazil since the late 1980s, aimed at reducing smoking rates^13^. Finally, our study identified higher proportions of former smokers among patients with specific cancer types, including lung, mouth, oropharyngeal, and stomach cancers. This aligns with Tseng et al. findings, suggesting greater success in quitting smoking among patients with tobacco-related cancers compared to those with non-tobacco-related cancers^26^.

Healthcare professionals providing care to cancer patients demonstrated a significant discrepancy in their engagement with smoking cessation interventions. Physicians were notably more inclined to implement such interventions, which include inquiring about tobacco use, assessing patients’ intentions to quit, offering guidance, and facilitating referrals for cessation support. This propensity can be attributed to the clinical training of physicians, which often emphasizes disease prevention and treatment, along with their responsibility to lead critical discussions about health behaviors that directly impact the efficacy of oncological treatment^27^ ^,^ ^28^. In contrast, nurses, while playing a fundamental role in patient care, appeared less proactive in initiating discussions regarding smoking cessation. This observation raises concerns about the training and support that nurses receive in relation to cessation interventions. Reduced training may hinder the participation of the entire healthcare team - including nurses, physiotherapists, and other allied professionals - in delivering effective smoking cessation treatment within the context of cancer care. As suggested by Matouq et al., there is an urgent need to enhance the knowledge and attitudes of healthcare professionals working in cancer treatment centers^29^.

Our study revealed that, although most healthcare professionals recognized the risks associated with tobacco use and provided guidance to their oncologic patients (74.9%), over half of them reported never discussing cessation strategies during consultations (52.9%). This gap in practice may be attributed to two primary factors, as reported in other studies, such as insufficient knowledge among health professionals regarding how to effectively support and implement tobacco cessation treatment and challenges in motivating cancer patients to quit smoking^29^ ^,^ ^30^ ^,^ ^31^. Despite these barriers, an overwhelming majority (95.6%) of healthcare professionals surveyed expressed the belief that smoking cessation should be an integral part of cancer treatment. Furthermore, these findings align with trends identified in previous studies, which have reported patterns of cessation counseling and prescription medication use among adult smokers. These studies underscore the importance of active physician involvement in positively influencing cessation rates^30^. The absence of similar initiatives from nurses may reflect an underutilization of the potential these professionals possess to impact patient health. It highlights the necessity for capacity-building strategies and encouragement for cessation interventions among all members of the healthcare team, as recommended by the U.S. Department of Health and Human Services^32^.

In addition, healthcare professionals play a pivotal role in patient identification and referral for treatment. However, studies have shown variations in the implementation of these practices in national cancer treatment referral centers, underscoring the need for a more comprehensive and integrated approach^10^ ^,^ ^12^. It is well established in the literature the importance of assessing health professionals’ knowledge of smoking, especially those who work in cancer treatment centers. Nonsmoker health professionals demonstrate more success in convincing patients to stop smoking^33^. However, they may lack the knowledge, skills, and confidence to offer smoking cessation interventions to cancer patients^9^. Furthermore, smoker patients who receive support and guidance from health professionals are more successful in quitting^34^.

In recent times, there has been substantial progress in making smoking cessation an essential component of cancer care for tobacco-using patients^35^. Emerging data continue to reinforce the notion that smoking cessation, even after a cancer diagnosis, significantly mitigates the harms associated with tobacco product use^36^ ^,^ ^37^ ^,^ ^38^. Moreover, a wealth of evidence guides us in understanding how to integrate tobacco treatment into the care of oncology patients and the difference it can make in managing tobacco-using patients^39^ ^,^ ^40^. Additionally, since 2017, the National Cancer Institute (NCI) has emphasized the importance of integrating smoking cessation interventions into cancer care at cancer treatment centers^36^. Despite strong evidence underscoring the importance of smoking cessation for cancer patients, it remains essential to ensure that healthcare professionals are able to deliver these interventions effectively. For patients who continue to smoke after a cancer diagnosis, evidence-based smoking cessation support should be integrated into multidisciplinary cancer treatment^41^. This approach helps patients maintain abstinence, improving treatment success rates and quality of life.

Although our study did not investigate the factors impacting smoking cessation among patients, previous research has shown that individuals with lower educational levels often face greater challenges in quitting, primarily due to limited access to information and cessation treatments^42^ ^,^ ^43^ ^,^ ^44^. Additionally, depressive symptoms and anxiety further hinder cessation efforts^45^ ^,^ ^46^ ^,^ ^47^. In contrast, physical activity has been identified as a facilitating factor for smoking cessation^47^ ^,^ ^48^.

This study has several limitations. First, its cross-sectional design prevents inferring causal relationships and limits the assessment of the long-term effects of smoking cessation interventions. Additionally, the non-probabilistic sampling of healthcare professionals may limit generalizability. Self-reported data, particularly regarding cessation practices, are susceptible to social desirability bias, especially in Brazil, where smoking oncologists face public stigma, potentially leading to underreporting, even in anonymous surveys. Missing data in some responses may have affected the robustness of certain analyses. The small number of smokers among healthcare professionals limited statistical power to compare groups. An additional limitation in the cancer patients’ group was the hybrid data collection approach, implemented due to the COVID-19 pandemic. Of the 210 cancer patients, 119 were interviewed in person and 91 via telemedicine. This variation may have affected response consistency.

In conclusion, our findings highlight the urgent need for enhanced training in tobacco cessation for healthcare professionals. The observed low confidence and engagement in delivering cessation support to cancer patients emphasize the need to prioritize this intervention in cancer care. Establishing effective strategies and fostering a cessation-oriented culture, especially in oncology centers, is essential. Tobacco use in cancer patients presents multifaceted challenges that impact survival, quality of life, and treatment efficacy. Prioritizing cessation as an integral part of oncology care is crucial for improving outcomes. Implementing robust strategies and offering continuous support throughout the cancer journey are essential steps toward better prognosis and well-being.
