# Supplementary material for: Perspectives on smoking cessation in the oncology environment: insights from brazilian patients and healthcare providers
Source: Rev Bras Epidemiol. 2025 Aug 8;28:e250046. doi: 10.1590/1980-549720250046 (PMC12333894; doi:10.1590/1980-549720250046)
Supplement: Supplementary file 1 [file 1980-5497-rbepid-28-e250046-sup.pdf]

Table S1 - Information on the tobacco use of cancer patients' group, such as age at smoking initiation, age at cessation and length of tobacco use, in addition to the smoking history of former and current smokers, level of nicotine dependence and motivation to stop smoking. BCH, Brazil, 2019 - 2021.

Scales	Variables	Relative frequency (%)	Absolute frequency (n)	n total
FAGERSTROM Level of nicotine dependence Ɨ (current smoking patients)	Low dependence	53.8%	14	26
	Moderate dependence	38.5%	10	
	High dependence	7.7%	2	
RICHMOND Motivation to quit smoking (current smoking patients)	Low motivation	18.5%	5	26
	Moderate motivation	7.4%	2	
	High motivation	74.1%	20	
HADS Hospital Anxiety and Depression Scale	With anxiety components	37.1%	78	210
	Without anxiety components	62.9%	132	
	With depression components	14.8%	31	210
	Without depression components	85.2%	179	

Ɨ Fagerstrom Test for Nicotine Dependence (Score: Low dependence 1-4; Moderate dependence 5-7; High dependence >8).

‡ The Richmond test is a 4-item instrument that evaluates motivation to quit smoking. Total score ranges between 0 and 10. Scores between 0 and 4 indicate low motivation; 5–6 moderate motivation; and 7–10 high motivation to quit.

Table S2 - Sociodemographic and tobacco use characteristics reported by healthcare professionals' group included in the study. BCH, Brazil, 2019.

Characteristic	Relative frequency (%)	Absolute frequency (n)	Total n
**Sociodemografic**			
Age			
21- 40 years old	76.0%	129	169
41 - 60 years old	24.0%	40	
Sex			
Female	58.0%	98	169
Male	42.0%	71	
Degree			
Medicine	50.3%	85	169
Nursing	12.4%	21	
Other□	37.3%	63	

Highest professional degree achieved				
	Technical	4.2%	7	167
	Bachelor's degree	23.4%	39	
	Specialization	47.3%	79	
	Master's degree	12.6%	21	
	Doctorate	10.2%	17	
	Other	2.4%	4	
Graduation				154
	≤ 10 years	84.4%	130	
	> 10 years	15.6%	24	
Time with patient				151
	< 50%			
	≥ 50%	34.4%	52	
		65.6%	99	
**Tobacco use**				
Smoking status				
	Current user	2.5%	3	122
	Former user	18.9%	23	
	Never user	78.7%	96	
E-cigarette				
	Current user	0.0%	0	
	Former user	12.5%	14	112
	Never user	87.5%	98	
Exposure to tobacco smoke in home				
	Yes	13.4%	16	121
	No	86.8%	105	
Exposure to tobacco smoke at work				
	Yes	3.3%	4	120
	No	96.7%	116	

Descriptive analysis

‡Others professionals include: psychologist, physiotherapist, nurse technician, auxiliary nurse, and radiotherapy technologist.

Missing data, not all the variables add to the total: Graduation (15); Time with patients (18); Smoking status (47); E-cigarette (57); Exposure to tobacco smoke in home (48); Exposure to tobacco smoke in home (49).
